# The impact of an innovative curriculum to introduce patient safety and quality improvement content

**DOI:** 10.1186/s12909-019-1604-0

**Published:** 2019-05-21

**Authors:** Kelly T. Gleason, Brigit VanGraafeiland, Yvonne Commodore-Mensah, Jo Walrath, Susan Immelt, Ellen Ray, Cheryl R. Dennison Himmelfarb

**Affiliations:** 1 School of Nursing, Johns Hopkins University, 525 N Wolfe Street, Baltimore, MD 21205 USA; 2 Carroll Health Group, Westminster, USA

## Abstract

**Background:**

The Fuld Fellows Program provides selected pre-licensure nursing students with a foundation in the science of patient safety, quality improvement and leadership through coursework and a mentored experience working on a quality improvement project. We evaluated this program’s impact on Fellows’ patient safety competence and systems thinking.

**Methods:**

Cohorts I-VI (*n* = 116) completed pre-post program evaluation that included measurement of patient safety competence through the Health Professional Education in Patient Safety Survey (H-PEPSS) and systems thinking using the Systems Thinking Scale. Pre- and post-program H-PEPSS and Systems Thinking Scale scores were compared using the Wilcoxon Signed-Rank Test. The Fellows were compared to non-Fellows on patient safety competence and systems thinking using t-tests.

**Results:**

Patient safety competence on all H-PEPSS scales improved from baseline to end of program: teamwork (2.6 to 3.1), communication (2.1 to 3.2), managing risk (2.2 to 3.3), human environment (2.8 to 3.7), recognize and respond to risk (2.7 to 3.6), and culture (2.9 to 3.8) (*p* < 0.05). The Fellows, in comparison to the non-Fellows, reported a significantly higher (*p* < 0.05) mean change score in five of the six H-PEPSS subscales. Fellows’ mean systems thinking score increased from 66 ± 7 at baseline to 70 ± 6 at program completion (*p* < 0.05), this mean post completion score was significantly higher than the non-Fellows reported mean STS score of 62 ± 7.

**Conclusion:**

The Fuld Fellows Program effectively facilitated patient safety and quality improvement and systems thinking learning among pre-licensure nursing students. This program can serve as a model for integrating quality and safety concepts into health professionals’ curricula.

## Background

In response to complex healthcare challenges in the United States, there is an urgent call for health professionals’ education to include development of patient safety and quality improvement competencies [1–3]. Despite the clear need, patient safety content continues to be insufficiently delivered across health professionals’ curricula [4, 5]. Adding patient safety content into curricula has several implementation challenges, including the lack of faculty prepared to teach such courses [6, 7], the limited available time in curricula [7, 8], and the increased amount of instructing done through simulation leaving less time to apply patient safety content in practical situations. The Essentials of Baccalaureate Education for Professional Nursing Practice state that nurses prepared in master’s nursing programs must develop skills to articulate methods, tools, performance measures, and standards related to quality, and be prepared to apply quality principles within an organization [9], highlighting the need for schools of nursing to meaningfully include quality improvement education in nursing curricula.

Nurses play key roles and demonstrate great success in leading efforts to improve quality of care and patient safety [10–15]. However, nursing students report a lack of understanding of patient safety principles and feeling incompetent in patient safety matters [16]. There is a paucity of nursing literature discussing a longitudinal curriculum for quality improvement and patient safety content in pre-licensure nursing curricula [17]. A review of patient safety learning identified that patient safety was often not clearly and explicitly taught as a subject in the nursing curricula [7, 16], and a lack evidence on which clinical learning environments facilitate the development of patient safety competencies in pre-licensure nursing students [18].

The Helene Fuld Leadership Program for the Advancement of Patient Care Quality and Safety (The Fuld Fellows Program) at the Johns Hopkins University School of Nursing is designed to address this gap in nursing education. The program prepares a select group of pre-licensure nursing students for future leadership at the bedside and in other care settings, who have exceptional competencies for promoting quality and safety. It focuses on the following objectives from the The Essentials of Baccalaureate Education for Professional Nursing Practice: 1) Demonstrate leadership and communication skills to effectively implement patient safety and quality improvement initiatives within the context of the interprofessional team, 2) Apply concepts of quality and safety using structure, process, and outcome measures to identify clinical questions and describe the process of changing current practice, and 3) Apply concepts of quality and safety using structure process, and outcomes measures to identify clinical questions and describe the process of changing current practice [9].

The purpose of this paper is to describe the Fuld Fellows Program and to answer the research question: Does participating in the Fuld Fellows Program increase Fellows’ perceived patient safety competencies and Systems Thinking? We hypothesized that Fellows’ perceived patient safety competencies and Systems Thinking would increase after participating in the Fuld Fellows Program, and that this increase would be significantly higher than students who did not participate. We also describe fellow and mentor satisfaction with the program.

## Methods

### Program description

The Fuld Fellows Program provides selected students with: 1) a broad, evidence-based, quality improvement and patient safety interprofessional education; 2) practical quality and patient safety learning experiences with Johns Hopkins improvement teams; and, 3) mentoring to bridge theory and practice. The program consists of 4 courses taken in parallel to the pre-licensure curriculum, a 3-day interprofessional intersession course, and a 6 month experience working with a quality improvement project (Table 1). Fellows who display a special interest in developing quality and safety skills beyond those standard curriculum are selected.

**Table 1:** Fuld Fellowship Curriculum

Semester	Course Content	Evaluations
Pre-Semester		Systems Thinking Scale
1	1) Overview of science of safety and an introduction to a culture of safety in healthcare 2) Explores enabling and contextual factors, including communication, teamwork, and human factors, that influence safety and quality 3) Introduces multiple methods to improve safety and quality, including Comprehensive Unit-Based Safety Program, disclosing adverse events, learning from defects, and patient-centered care 4) Translating evidence into practice/leading change 5) Capacity building for improved patient safety and quality	
2	1) Experiential learning working on a mentored quality improvement project (50 h a semester for 2 semesters) 2) Monthly seminar to review and reinforce key patient safety & quality concepts	
3		
Post-Semester 3		Mentor/Mentee Satisfaction
4	1) Leadership to improve safety in complex systems 2) Advocacy for patients, families, and colleagues 3) Prepare and present scientific poster disseminating results of mentored quality improvement project	
Post-Semester 4		Focus group, Systems Thinking Scale, and Health Professional Education in Patient Safety Survey
1 Year Post-Fellowship		Health Professional Education in Patient Safety Survey

The first semester course applies a systems approach introducing the Fellows to the science of patient safety and quality improvement. Using case studies, case-based quizzes and peer assessments, the course prepares Fellows with basic knowledge and skills necessary for their work with a clinical project team. Fellows work 50-h per semester in semesters two and three with a mentor and interprofessional, patient safety or quality improvement team in a Johns Hopkins clinical setting. In addition, they participate in bi-weekly, online reflective learning journals focusing on specific attributes of safe, high reliability healthcare delivery teams. In the fourth and final semester, Fellows create a scholarly poster presentation and disseminate their project-based learning. The coursework in this final semester focuses on providing realistic and practical approaches for translating Fuld learning in quality and safety into the clinical work settings the Fellows are preparing to enter. Fellows have an opportunity to participate in a 3-day interprofessional patient safety simulation course that includes nursing and medical students taught by interprofessional faculty. This course focuses on improving students’ teamwork and communication skills and system-based thinking and addresses the causes of preventable harm and evidence-based strategies for harm prevention. Cohorts I-VI (*n* = 115) have completed the program and evaluations as of December 2015. The Fuld Fellows Program continues and has been adapted for delivery in the new Master’s Entry to Nursing Program is currently educating the twelfth cohort of students. Institutional Review Board approval (00087543) was obtained to evaluate the effectiveness of the program.

### Evaluation

Process evaluation includes specific questions about course content, delivery, and attainment of learning goals for each course and at the end of program. Project mentor and Fellow evaluate each other in the middle and at the end of the program. Focus groups are conducted at the completion of the program. Pre-post program evaluation includes Fellows’ perceptions of patient safety competence at entry into practice, using the Health Professional Education in Patient Safety Survey (H-PEPSS), and systems thinking, using the Systems Thinking Scale (STS) [19]. The pre-licensure nursing students who did not participate in the Fuld Fellows Program also complete the H-PEPSS survey and STS at the completion of the program to allow for post-program comparison across groups.

H-PEPSS measures self-reported patient safety competence through focusing on six domains of safety competencies: culture of safety, working in teams with other healthcare providers, effective communication, managing risk, optimizing human and environmental factors, and recognizing and responding to adverse events to measure patient safety learning [20]. H-PEPSS consists of 16 items and responses are measured in a 5-point disagree-agree Likert type scale. The items begin with ‘I feel confident in what I learned about … ’ and include a ‘don’t know’ option. Previous studies assessed the H-PEPSS validity and found that the Cronbach’s alpha score ranges from 0.81 to 0.85 for all six domains [20]. For each item, the participants were asked to separately respond with their level of agreement before the program and after completion of the program.

The STS is a 20-item instrument that evaluates participants’ systems thinking in quality improvement work, such as “I think recurring patterns are more important than any one specific event” and “I consider the relationships among co-workers in the work unit.” The STS measures items on a 5-point Likert-type scale and are scored 0 to 4. The total score can range from 0 to 80 and is created by calculating the sum of the responses for each item. The STS is a validated scale with a Cronbach’s alpha score of 0.89 [19].

A quasi-experimental design was used to compare Fellows to students who were not involved in the program for cohorts III through V. The Fellows in cohorts III through V were compared to their colleagues on outcomes of patient safety competence and systems thinking. This evaluation occurred at the time of their graduation from the pre-licensure nursing program.

Stata version 15 (StataCorp LP, College Station, TX was used for all analyses. Pre- and post- program scores for the H-PEPSS subscales and STS were compared using the Wilcoxon match-pairs signed-rank test for the participants who completed both the pre- and post-surveys. Pre-Post change scores were calculated and mean change scores for Fuld Fellows and non-Fuld Fellows were compared using t-tests. The Fuld Fellows and non-Fuld Fellows’ post-program STS scores were compared using t-tests. Student and mentor satisfaction with the program was analyzed through descriptive statistics.

## Results

The majority of the Fellows from Cohorts I-VI were female (87%) and between the ages of 23–29 (81%) (Table 2). The highest degree earned prior to entry to the program was a Bachelor’s for the majority of the Fellows (81%). The three most common pre-nursing fields of study were biology and health sciences (34%), psychology (15%), and anthropology (8%). Thirty percent of the Fellows reported prior participation in patient safety projects. Most of the mentors had at least a Masters degree and held positions of leadership at Johns Hopkins. The setting of the projects varied significantly (Table 3), which gave the Fellows an opportunity to choose projects best-suited to their interests.

**Table 2:** Description of Fuld Fellows

	Participants (Cohorts I-VI)
	*N* = 116
Sex	
Female	89% (104)
Age at program entry	
22–29	84% (98)
30–39	12% (14)
40–49	4% (5)
Highest Degree	
Bachelor’s	83% (96)
Masters	15% (18)
JD	1% (1)
PhD	1% (1)
Pre-Nursing Field of Study	
Biology and health sciences	31% (36)
Psychology	16% (19)
Anthropology	7% (8)
Sociology	4% (5)
Other	41% (48)

**Table 3:** Examples of Mentors and Projects

Project Title	Project Setting	Project Category	Mentor Role
Perioperative Anti-Platelet and Anticoagulation Management Task Force Targeted at reducing readmissions in the Department of Medicine	Peri-operative care	Readmission reduction	Professor of Anesthesiology/Critical Care Medicine
Nursing Role and Work Flow Assessment	Inpatient	Safe Nursing Practice	Senior Director of Nursing of Ambulatory Services
Rapid Access to Tertiary Care: A Clinical, Financial and Operational Model	Emergency care	Disaster care	Assistant Director of Nursing
Early Sepsis Management	Intensive Care Unit	Sepsis management	Clinical Nurse Specialist
Prevention for Positives (Healthy Prevention Behaviors for HIV Positive Patients)	Outpatient	Health Prevention	Nurse Manager
Reducing Avoidable Hospitalizations	Outpatient	Readmission reduction	VP/COO & Sr. Director Quality/Safety, Johns Hopkins Home Care Group
Implementing the Central Line Checklist in the Operating Room	Operating Room	Infection prevention	Director of Nursing
Reducing length of stay for Hospitalist patients	Inpatient	LOS reduction	Senior Director of Quality
Evolution in postoperative Pain management	Surgery	Pain management	Associate professor of anesthesiology and critical care
Implementation of a Transitional care unit for the Department of Neurosciences	Post Anesthesia Care Unit	Implementation	Director of Nursing
Investigation of Potential Safety Issues with Acetaminophen use in Surgical Patients	Surgery	Medication Safety	Education Lead, Department of Surgery

### Perceptions of patient safety competencies

The Fellows in cohorts II through VI completed the H-PEPPS survey (*n* = 52). The Fellows’ mean scores on the H-PEPSS scale improved from baseline to the completion of the program. Mean deviation scores on the H-PEPSS scales improved from baseline to end of program as follows: teamwork (2.6 to 3.1), communication (2.1 to 3.2), managing risk (2.2 to 3.3), human environment (2.8 to 3.7), recognize and respond to risk (2.7 to 3.6), and culture (2.9 to 3.8) (*p* < 0.001 for all). Mean deviation scores on the H-PEPSS scales continued to improve 12 months following the program. Compared to immediate post-program scores, there was an increase in teamwork (1.0), communication (1.0), managing risk (0.8), human environment (0.9), recognize and respond to risk (0.7), and culture (0.7). The increase in recognize and respond to risk and culture was significant (*p* < 0.05).

### Systems thinking in quality improvement

The Fellows in cohorts I and III through VI completed the STS (*n* = 65). The mean scores on the STS improved from baseline to completion of the program in each of the cohorts. Mean ± standard deviation scores on the systems thinking scale increased from 66.1 ± 7.1 at baseline to 70.3 ± 6.4 (*p* < 0.001).

### Comparison to non-fellows

**Fig. 1:**
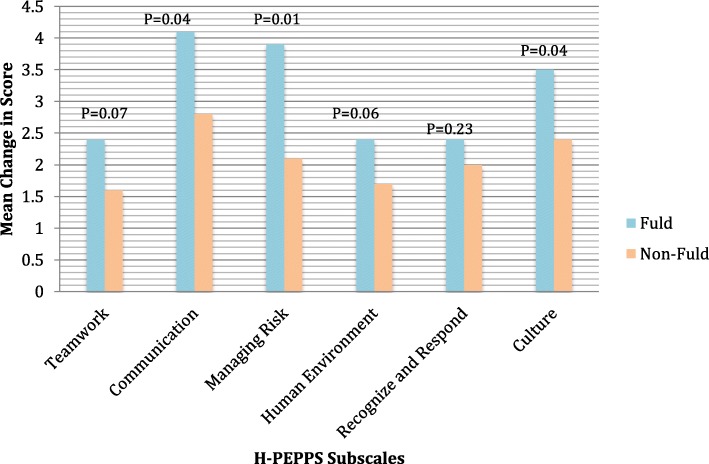
Patient Safety Competency at Entry into Practice: Comparing Mean Change for Fuld and Non-Fuld Students

**Fig. 2:**
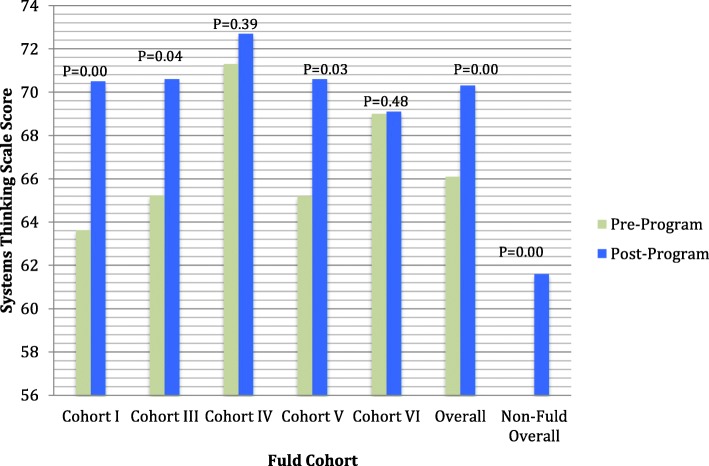
Results of System Thinking Scale

Both the Fellows (*n* = 36) and non-Fellows (*n* = 37) who participated in the evaluation reported a significant increase (*p* < 0.001) across all six domains of patient safety competencies. The Fellows reported a significantly higher (*p* < 0.05) mean change score in comparison to the non-Fellows in five of the six domains of safety competencies: effective communication, working in teams with other healthcare providers, optimizing human and environmental factors, and culture of safety (Fig. 1). The Fellows reported a higher, but not statistically significant, mean change score in the domain of recognizing and responding to adverse events. The Fellows reported a mean STS score of 70.73 ± 5.96; this score was significantly higher (*p* < 0.001) than the non-Fellows reported mean STS score of 61.63 ± 7.43 (Fig. 2).

### Fellow and Mentor evaluations

**Fig. 3:**
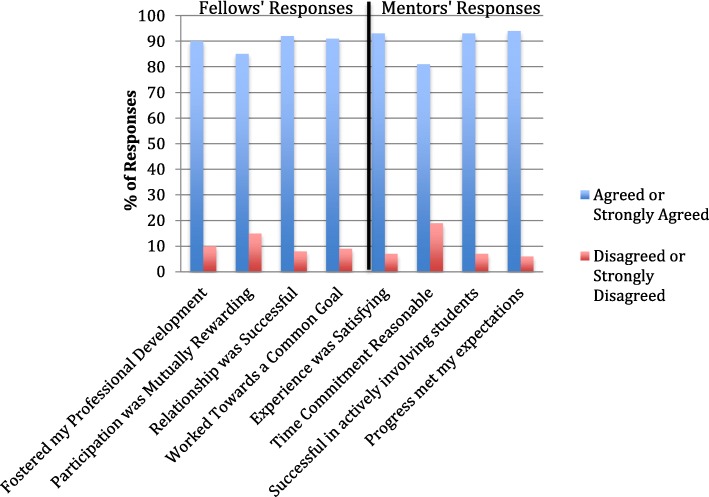
Mentor and Fellow Satisfaction with Relationship

The results of the evaluations indicate that most Fellows and mentors found their relationship rewarding (Fig. 3). Also, 92% of the Fellows agreed or strongly agreed that the relationship was successful, and 90% agreed and or strongly agreed that their mentors fostered their professional development. Furthermore, 93% of the mentors who completed the evaluations agreed or strongly agreed that the experience was satisfying and 94% agreed or strongly agreed that the Fellows’ progress in meeting the agreed upon the project goals met their expectations.

### Focus group results

In the focus groups, the Fellows emphasized the value of exposure to patient safety and quality improvement education and hands-on projects. One Fellow stated: “I believe I will be more aware of quality improvement and safety issues and this will make me a leader in the workforce.” Fellows who participated in the interprofessional education opportunity reported that it was a highlight of their Bachelor of Science in Nursing program and that they felt well-prepared to contribute to the interprofessional quality improvement case studies. Fellows gave feedback that they were excited to apply their knowledge as they embarked on their nursing careers and planned to be involved in patient safety and quality improvement projects in their future workplaces.

## Discussion

Educating health professionals on the fundamentals of patient safety and quality improvement is essential to advancing a culture of safety within an increasingly complex and dynamic health care system [1]. The Fuld Fellows Program addresses the need for this education through: 1) didactic classes throughout the program dedicated to patient safety and quality improvement concepts, 2) an academic-clinical partnership that provides the Fellows with an opportunity to work in a clinical setting with a quality improvement team and, 3) an interprofessional course. The Fellows’ perceived patient safety competencies and Systems Thinking significantly increased after participating in the program, and this increase was greater than students who did not participate. This program serves as a model for meaningfully including patient safety content in health professionals’ education.

The Fuld Fellows Program runs parallel to the pre-licensure program, which provides Fellows with continuing content of patient safety concepts as their learning progresses. Previous work showed nursing students’ lack of self-reported confidence in their knowledge of patient safety [16, 17]. The ongoing connections built between safety concepts and practice through journaling and discussion with mentors and faculty may explain the improvement in perceptions of patient safety competence and in systems thinking across the four semesters.

Students’ confidence in their understanding of patient safety declines as they advance in their academic program and become more exposed to the complexities of health care [21]. Results of a study where the HPEPSS was administered annually over 4 years suggest that confidence in the clinical dimensions of patient safety improve in the first 2 years of nursing school then decline in the second 2 years [21]. The authors also found that students’ confidence in speaking up about patient safety issues declined as they gained experience in clinical settings. There is a great need for nurses to enter into practice prepared to professionally communicate about potential safety risks [22]. We hypothesized that Fellows’ ongoing connections with Fuld faculty and with supportive mentors in leadership patient safety positions may explain why their confidence not only did not decrease, but actually increased, as shown by the results from the same instrument (HPEPSS). Qualitative results of focus groups confirm this impression. The Fellows perceived the Fuld Fellows Program as a distinct part of their experience at Johns Hopkins. The experiential learning enabled the Fellows to understand the challenges of implementing change in real clinical settings. Several Fellows described their participation in the Fuld Fellows Program as an advantage over non-participants. In addition, they saw their relationships with their mentors as supportive and contributory to their abilities to continue as leaders in patient safety following graduation. This was the goal of the Fuld Fellows Program.

Interprofessional education is required by licensing bodies of health professions and this includes understanding the roles and responsibilities of both professions, engaging in effective communication, collaborating around shared ethics and values, and engaging in teamwork [6, 23, 24]. The Fellows engaged in an interprofessional training program that focused on improving students’ interprofessional teamwork, communication skills and system-based thinking [25]. The interprofessional education component may have additionally contributed to the improved perception of patient safety competency.

Our findings are limited by the small size of the combined Fellow cohorts (*N* = 116) and the fact that it was conducted at a single academic medical system with a Patient Safety Institute. There was no objective measure of the students’ patient safety and quality improvement learning. Furthermore, the Fuld Fellows were selected based on their commitment to patient safety and quality improvement, thus there is selection bias in comparing Fuld and non-Fuld Fellows. It is important to note that eligible students opted not to do Fuld because there were limits on how many extracurricular activities students could partake in, thus non-Fuld Fellows were exposed to other learning opportunities during this time that did not increase their systems thinking and patient safety competencies compared to Fuld Fellows. While Fuld Fellows demonstrated a continued increased in patient safety competencies 1 year following completion of the program, we did not assess non-Fuld Fellows patient safety competencies at the 1 year time point and thus cannot compare the sustained increase. A measure that specifically addresses the role of training in patient safety learning in health professionals’ education, such as the Short Version of the Attitudes to Patient Safety Questionnaire [26], may be important to future evaluations of training in patient safety competencies.

Although the curriculum and teaching methodologies for most of the program can be replicated, it may be challenging to find the number and range of knowledgeable and willing mentors in other locations. Faculty time is required as well, one faculty member served as the Fuld faculty for each group of Fellows. The Fuld Faculty had education and training in patient safety and experience in the clinical setting. Responsibilities included instructing the one-credit seminar, and overseeing the mentor-mentee relationship. There were occasional challenges with identifying the right mentor and mentee match, and ensuring that both the Fellow and the mentor were satisfied with the Fellow’s level of commitment and time spent on the project. The Fuld faculty helped resolve these issues and assure accountability by making site visits, keeping in touch with mentors by phone or email, solving problems, and meeting with the Fellows in a classroom setting once a month. The selectivity and cohort nature of the Fuld Program also contributed to its success. The Fellows were selected for their interest in patient safety, so their increasing sense of efficacy and positive relationships with mentors was in some part explained by their motivation to apply to the program in the first place.

Further work on programs in health professionals’ education specifically focused on patient safety and quality improvement is ongoing and worthwhile. Part of that effort has to be refining and improving evaluation of pre-licensure patient safety education and its impact on health professionals’ postgraduate participation and leadership. As the literature grows, faculty will find more evaluation instruments with strong psychometrics to guide streamlining existing program.

## Conclusion

The Fuld Fellows Program effectively introduces a select group of pre-licensure nursing students to essential patient safety and quality improvement concepts. Though all students in the pre-licensure nursing program reported increased patient safety competence, the Fellows reported higher levels of systems thinking and greater improvement in patient safety competence after participation in the program than the pre-licensure students who did not take part in the program. This program can strengthen health professionals’ education by offering an academic model and innovative curriculum for building competencies in quality and safety that can be adapted at other institutions.
